# News and Events of the Week

**Published:** 1910-02-05

**Authors:** 


### News and Events of the Week.

Dr. F. H. Champneys has been re-elected a representative of the R.C.P. on the Central Midwives Board.

Mr. W. H. Lever has been appointed Chairman of the Liverpool School of Tropical Medicine, in place of the late Sir Alfred Jones.

The Central Midwives Board has unanimously passed a resolution deeply regretting the death of Dr. Stanley Atkinson and sympathising with his family.

At the London University regulations have been adopted to establish "Dental Surgery" as a second branch in which the degree of Master of Surgery can be obtained. The examination in this, as in the existing branch, will be open only to candidates who have taken the M.B., B.S. degrees.

Mr. A. G. R. Foulerton, F.R.C.S., will deliver the Milroy Lectures on February 17, 22, and 24; Dr. J. S. Bolton the Goulstonian on March 1, 3, and 8; and Dr. W. Osler, Regius Professor of Medicine in the University of Oxford, the Lumleian Lectures on the 10th, 15th, and 17th of the same month.

The Romany Amateur Dramatic Club is giving two performances of "Captain Brassbound's Conversion," by Bernard Shaw, at the Court Theatre, on Monday and Tuesday evenings, February 7 and 8, in aid of the fund for the removal of King's College Hospital to South London. Tickets are to be had of Mr. George Heyer, Appeal Secretary, King's College Hospital, W.C.

The Belgian expedition which will leave shortly for the Congo to make investigations in connection with sleeping sickness will be in charge of Dr. Rhodain, Professor of Bacteriology at the University of Louvain. Work will be begun in the northern part of the Katanga district. The expedition will go to Bukama, in the neighbourhood of the Kalengwe Falls, on the Lualaba River.

At the ordinary quarterly *comitia* of the Royal College of Physicians of London, the President, Sir R. Douglas Powell, being in the chair, the following Fellows of the College: Dr. Edward Liveing, Dr. W. C. Donald Hood, Dr. H. W. G. Mackenzie, and Sir James Reid, K.C.B., M.D., were elected councillors, in place of Sir William Allchin, M.D., Dr. T. R. Glynn, Dr. G. N. Pitt, and Dr. R. P. Smith.

Calling attention to the Congress on the Administrative Sciences to be held in Brussels next July, Sir George Fordham, Chairman of the British Committee, moved that a set of the Board's publications of the Central Midwives Board should be placed at the disposal of the British Committee. This was carried unanimously. Sir George has prepared a history of the work of the Board, in French, for the Congress.

The following institutions have been added to the list recognised by the examining board of the R.C.P. in England: The Royal Free Hospital, the London and Edinburgh Schools of Medicine for Women, to the list of medical schools; the Mater Infirmorum Hospital, Belfast, for Clinical Instruction in Medicine and Surgery, in connection with Queen's College, Belfast; and the laboratories of the Royal Colleges of Physicians and Surgeons of Edinburgh, for the course of instruction in public health.

Dr. William J. Robinson, editor of the *Critic and Guide*, the *American Journal of Urology* and *Therapeutic Medicine*, has purchased the Chicago *Clinic*, which has had an uninterrupted existence for 23 years (though known during the past year under the title of *Practical Therapeutics*), and has consolidated it with *Therapeutic Medicine*. The consolidated journal will be published monthly.

At the meeting of Convocation of the Manchester University recently, the question of appointing qualified medical women to resident posts in the Royal Infirmary was the subject of an important debate. At the close the following resolution was carried by a large majority: "That, having regard to the interests of the women students in the Faculty of Medicine, Convocation desires to suggest to the University Court that they should ask the Royal Infirmary Board to reconsider the question of appointing women to resident posts at the Royal Infirmary."

On Saturday, January 15, there were 125,881 paupers relieved, of whom 81,741 were indoor and 44,140 outdoor. Compared with the corresponding week of 1909 these figures show a decrease of 564 indoor poor and a decrease of 4,125 outdoor poor, a gross decrease of 4,689; with 1908, an increase of 562 indoor poor and a decrease of 4,271 outdoor poor, a net decrease of 3,709; with 1907, an increase of 2,778 indoor poor and a decrease of 875 outdoor poor, a net increase of 1,903. The casuals numbered 1,025, as against 964 in the previous week. Of the children under sixteen years of age, 764 were boarded out beyond the limits of the union or parish.

The Holborn Borough Council has decided to abandon a proposal that the Local Government Board should be urged to allow the Metropolitan Asylums Board to use a vacant isolation hospital at Gore Farm for sanatorium treatment of consumptive patients. The Public Health Committee then presented a report on proposed further measures for the prevention of phthisis. The M.O.H. for Holborn recently reported that in 1903-5 its death rate from phthisis was the highest in London. Phthisis was very general among the inmates of common lodging-houses. The Council had already provided for the voluntary notification of the disease; and it was resolved to convene a conference of local philanthropic workers to consider the possibility of further measures.

A Sessional Meeting of the Royal Sanitary Institute will be held at 90 Buckingham Palace Road, on Wednesday, February 9, at 8 p.m., when a paper will be read on "The Ventilation of the Large Examination Hall, Cambridge," by the Rev. J. B. Lock, followed by a discussion on "The Ventilation and Warming of Public Buildings," in which the following have consented to take part: Henry Adams, M.Inst.C.E., T. W. Aldwinckle, F.R.I.B.A., James Kerr, M.A., M.D., D.P.H., Louis C. Parkes, M.D., D.P.H., E. Thomas Swinson, and Alfred E. Wheeler. The Henry Saxon Snell prize for 1909, awarded to Mr. Alfred E. Wheeler, and supplementary prizes awarded to Mr. J. Roger Preston, M.R.San.I., and Mr. E. Thomas Swinson, M.R.San.I., will be presented at the opening of the meeting. Tickets for admission of visitors may be had on application to the secretary.

